# An Intronic cis-Regulatory Element Is Crucial for the Alpha Tubulin *Pl-Tuba1a* Gene Activation in the Ciliary Band and Animal Pole Neurogenic Domains during Sea Urchin Development

**DOI:** 10.1371/journal.pone.0170969

**Published:** 2017-01-31

**Authors:** Salvatore Costa, Aldo Nicosia, Angela Cuttitta, Fabrizio Gianguzza, Maria Antonietta Ragusa

**Affiliations:** 1 Department of Biological, Chemical, and Pharmaceutical Sciences and Technologies, University of Palermo, Palermo, Italy; 2 Laboratory of Molecular Ecology and Biotechnology, National Research Council-Institute for Marine and Coastal Environment (IAMC-CNR) Detached Unit of Capo Granitola, Torretta Granitola, Trapani, Italy; Laboratoire de Biologie du Développement de Villefranche-sur-Mer, FRANCE

## Abstract

In sea urchin development, structures derived from neurogenic territory control the swimming and feeding responses of the pluteus as well as the process of metamorphosis. We have previously isolated an alpha tubulin family member of *Paracentrotus lividus* (*Pl-Tuba1a*, formerly known as *Pl-Talpha2*) that is specifically expressed in the ciliary band and animal pole neurogenic domains of the sea urchin embryo. In order to identify *cis*-regulatory elements controlling its spatio-temporal expression, we conducted gene transfer experiments, transgene deletions and site specific mutagenesis. Thus, a genomic region of about 2.6 Kb of *Pl-Tuba1a*, containing four Interspecifically Conserved Regions (ICRs), was identified as responsible for proper gene expression. An enhancer role was ascribed to ICR1 and ICR2, while ICR3 exerted a pivotal role in basal expression, restricting Tuba1a expression to the proper territories of the embryo. Additionally, the mutation of the forkhead box consensus sequence binding site in ICR3 prevented *Pl-Tuba1a* expression.

## Introduction

*Cis*-regulatory elements, such as promoters, enhancers and silencers, are involved in determining the spatio-temporal patterns of gene expression. Accordingly, the precise identification of these elements is an essential step in understanding the regulatory networks operating at the cellular, tissue or organismal level [1]. It is also known that the *cis*-regulatory system may be separated into several *cis*-regulatory modules, whose topological integration establishes a specific pattern of each gene expression and orchestrates the dynamic regulation of gene expression of an embryonic domain in response to environmental and developmental stimuli [2–4]. The Gene Regulatory Networks involved in each specification step of differentiation processes have been extensively studied in echinoderms [5–9]. In particular, the neural differentiation process of sea urchin embryos has been thoroughly analysed [10–15].

All echinoderm larvae possess a nervous system consisting of a ciliary band and associated sensory ganglia (apical, oral and lateral ganglia) that control swimming and feeding [16–21]. Neurons of the larval nervous system of euechinoids first appear as neuroblasts in the thickened ectoderm of the animal plate (anterior neuroectoderm, ANE) at the late blastula–early gastrula stage. Neurons continue to be added throughout larval development, and the fully formed eight-armed pluteus nervous system is organized as a complex array of sensory neurons, interneurons, tracts of axons and ganglia that are closely associated with the larval ciliary band neuroectoderm (CBE) and larval muscles [13].

We have previously identified and characterized a specific alpha tubulin gene (*Pl-Tuba1a*, formerly named *Pl-Talpha2*) in the *Paracentrotus lividus* sea urchin whose expression begins at the hatching blastula stage and is restricted in the major structures that will give rise to the larval nervous system [9, 22–24]. Interestingly, in the same territories is also specifically expressed a beta tubulin gene [25] encoding an isotype containing a carboxy terminal domain that is typical of neural specific tubulin isoforms. Gene transfer experiments showed that a *Pl-Tuba1a* 5.3 Kb genomic region is involved in the specific temporal and spatial regulation of this gene [26]. Moreover, mechanisms of epigenetic modifications contributing to its expression during embryo development were characterized [27].

Previously, we have identified several putative Interspecific Conserved Regions (ICRs) using computational techniques [26]. In this work, we identify a genomic region of about 2.6 Kb of *Pl-Tuba1a*, containing four ICRs, as responsible for proper gene expression. In addition, an overall analysis of the ICRs was performed by the gene transfer of properly modified reporter constructs, revealing their role in the regulation of *Pl-Tuba1a* gene expression.

## Materials and Methods

### Preparation of reporter constructs

The 5’ deletion constructs were generated by PCR amplification of the full-length clone (Pl-Tuba1a-GFP [26] using appropriate HindIII primer sets (see S1 Table) and subsequent cloning into the HindIII site of pBluescript II SK(+) (pBSK) vector (Stratagene). The GFP reporter constructs maintain the GFP coding sequence in frame with the first three codons of *Pl-Tuba1a* and are under the control of progressively reduced amounts of *Pl-Tuba1a* regulatory sequences.

Internal (ICR3 and/or ICR4) deletions were generated by PCR amplification of the -1.8KbGFP construct, excluding each conserved region, using the appropriate primer set and subsequent self-ligation of the two PCR products, permitted by XbaI restriction sites harboured by primers, and cloning into the HindIII site of pBSK vector.

The -1.8(ΔIntron) was obtained by PCR amplifications of the -1.8KbGFP construct, excluding the first intron, using the appropriate primer sets and subsequent self-ligation of the two PCR products, exploiting a KpnI restriction site neighboring the 5’ end of the GFP coding sequence and putting in frame the first three codons of *Pl-Tuba1a* with GFP ORF.

All the corresponding Luc clones were prepared by replacing the GFP coding sequence via KpnI digestion, with the Luc coding sequence amplified from pXP1 plasmid (ATCC) with a proper 5’ KpnI modified primer set.

All the PCR amplifications were performed using Phusion High-Fidelity DNA Polymerase (Thermo Fisher Scientific), and resultant clones were sequenced to confirm correct insertion and frame maintenance.

The -1.8 Mutant clone was obtained via the QuickChange II Site-Directed Mutagenesis kit, following the manufacturer’s instructions (Agilent Technologies). The -1.8Kb clone was used as a DNA template with the primer set indicated in S1 Table.

### Microinjection of constructs and reporter analysis

Sea urchin eggs were injected with 2 pl of a solution containing 5 ng/μl of linearized plasmid (GFP or Luc reporter) together with 5% Texas Red-conjugated dextran, 25 ng/μl carrier DNA (prepared by enzymatic digestion of *P*. *lividus* sperm DNA size selected to average length of 5 to 10 Kb), 1M KCl, and 20% glycerol, following the microinjection and embryo culture procedures previously described [23, 28, 29]. Each construct was microinjected at least in triplicate (almost 300 embryos microinjected/experiment) using different batches of sea urchin eggs. As negative controls, pBSK vectors containing GFP or Luc coding sequences were used.

At the desired developmental stage, injected embryos were harvested and, in the case of GFP reporter injected embryos, mounted on glass slides and examined under an epifluorescence Olympus BX50 microscope. Bright-field, GFP fluorescence, and Texas Red fluorescence images were captured with a Nikon digital camera and processed using the Nikon Nis-Elements software. Statistical analysis of expression patterns performed at the pluteus stage (where neurogenic territories are easily identified) showed GFP proper localization in approximately 90% of injected embryos, reflecting the way that exogenous DNA is incorporated in a mosaic fashion.

Luc assay was performed injecting about 300 eggs for every reporter construct. Embryos, immediately after reaching the gastrula stage, were preventively checked for Texas Red staining, counted and recovered in presence of CCLR lysis buffer at the ratio of 1μl to 1 injected embryo. Lysis was than executed as indicated in the Luciferase Assay System from the Promega Corporation. Samples corresponding to 5 μl of each lysate were read for light intensity after the addition of 100 μl of the Luciferase Assay Reagent included in the kit, with a typical delay time of 2 seconds and a read time of 10 seconds on a Promega GloMax-Multi Detection System. Each experiment was repeated in triplicate.

## Results

### Identification of promoter regulatory elements

In order to identify the minimum regulatory regions necessary and sufficient to drive the proper enhanced spatio/temporal expression of the *Pl-Tuba1a* gene (*GenBank*: *JF272003*), several GFP reporter constructs were generated starting from the recombinant full construct (Pl-Tuba1a-GFP) containing a genomic region from -4.5 Kb to +0.8 Kb. This genomic region contains the transcription start site (TSS), the first exon containing the 5’ UTR and including the ATG, the first intron and two codons of the second exon. Therefore, the construct encodes a fusion protein containing the amino terminal three alpha tubulin amino acids fused to GFP [26]. A series of 5’ shortened constructs was generated and used in gene transfer analysis assays (see S1 Fig in supplementary materials).

**Fig 1:**
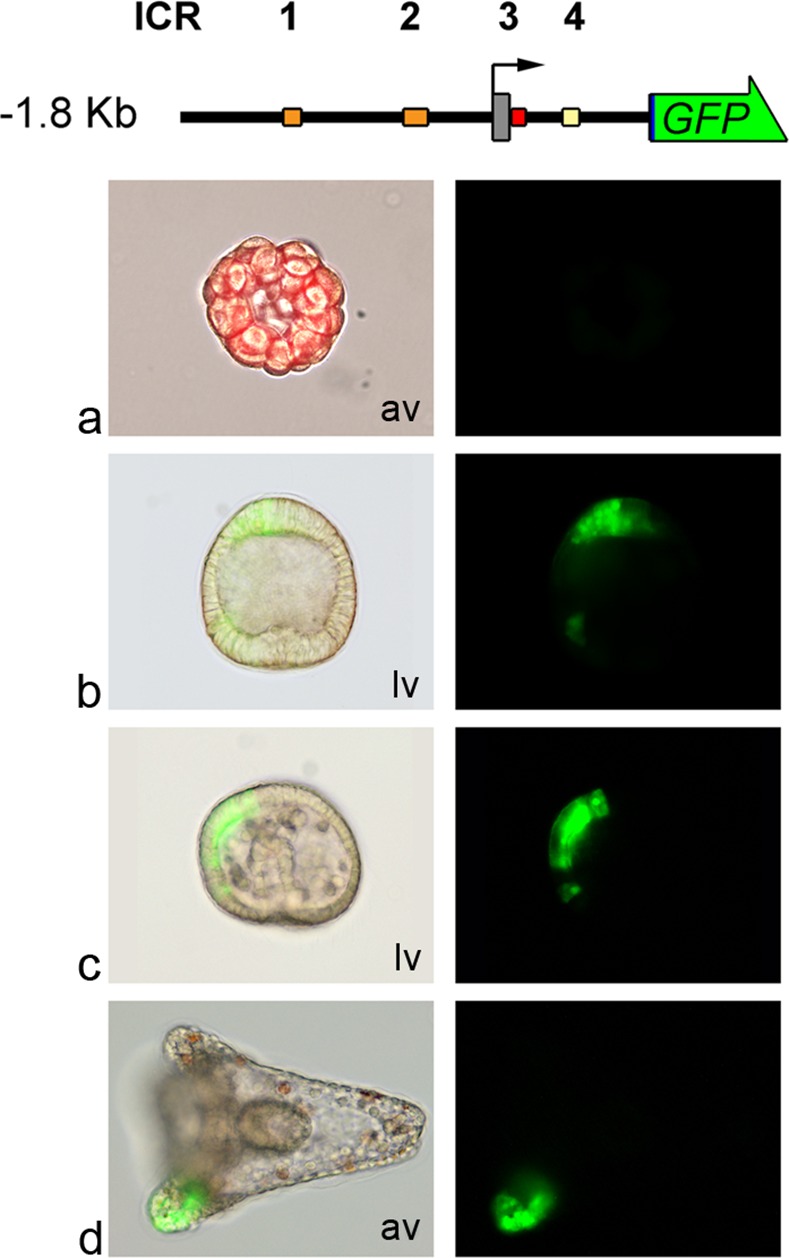
Expression of the -1.8KbGFP transgene construct during *P*. *lividus* embryo development. At the top is a schematic structure (drawn to scale) of the Pl-Tuba1a -1.8KbGFP reporter construct. The bent arrow indicates the TSS. A grey box represents the first exon (5’UTR and ATG start codon). Downstream of the first exon there are the first intron and two codons of the second exon. Coloured boxes indicate the four ICRs. For sake of simplicity, only the section of ICR3 inside the intron is shown. The arrowed green box represents the GFP reporter gene cloned in frame with the alpha tubulin codons. Left: triple-merged images (bright-field, GFP fluorescence and Texas Red fluorescence-a) or merged fluorescence and bright-field images (b, c, d). Right: GFP fluorescence images from microinjected embryos. × 20 magnification. (a) 32-cell stage; (b) Blastula stage; (c) Gastrula stage; (d) Pluteus stage. Lv: lateral view; av: animal view.

Using this approach, we found that the genomic sequences included between -1.8 Kb and +0.8 Kb (the -1.8KbGFP construct) are still able to drive the proper spatio-temporal expression of the Tub-GFP fusion protein. Indeed, GFP expression was detected from the blastula to pluteus stages and was strictly localized in the animal pole and ciliary band domains of the embryos, as previously described for the Pl-Tuba1a-GFP transgene [26]. Fig 1 shows the results of the gene transfer experiments performed with the -1.8Kb GFP construct herein selected as reference for the successive experiments and as template to produce further clones.

Interestingly, this region contains four of the six ICRs which were previously identified by an *in silico* approach. Two of them, ICR1 (approximately 100 bp, between -1200 and -1100 bp) and ICR2 (130 bp, between -510 and -380 bp), are located in the 5’ upstream region; ICR3 (about 240 bp, located between -50 and + 190 bp) contains the TATA box, the transcript leader sequence and a portion of the first intron sequence; ICR4 (located between +400 and +500 bp) is contained in the first intron [26].

### Functional analysis of interspecific conserved regions located upstream of the TSS

In order to understand the functional role of each ICR, we performed additional gene transfer experiments; thus, other constructs lacking ICR1 or ICR1-2 were microinjected. These constructs provided similar GFP expression patterns, and results are reported in S1 Fig.

GFP expression was properly detected from the blastula stage (data not shown) to the pluteus stage, and its expression was restricted to the proper territories. Therefore, their removal does not affect spatio/temporal regulation. However, it appears that a progressive reduction of GFP expression occurred after each ICR deletion with respect to the -1.8KbGFP transgene.

**Fig 2:**
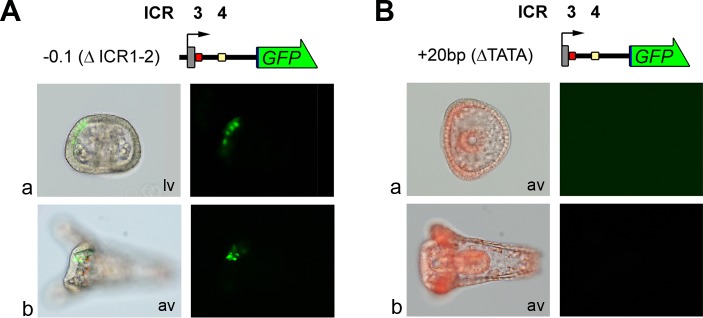
Transgene basal expression and loss of expression by TATA box deletion. Gene transfer assay results performed using the -0.1(ΔICR1-2)GFP (A) and the +20(ΔTATA)GFP (B) constructs. Structure and conventions are the same as in Fig 1. (a) Gastrula stage; (b) Pluteus stage. (A) Merged fluorescence and bright-field images (left) and GFP fluorescence images (right) from microinjected embryos are shown. (B) Triple-merged bright-field, GFP fluorescence and Texas Red fluorescence images (left) proving embryos are microinjected and GFP fluorescence images (right). × 20 magnification. Lv: lateral view; av: animal view.

Further deletions, including -0.1(ΔICR1-2)GFP which contains solely the TATA box and the first intron, did not result in any change in spatio/temporal regulation (Fig 2A).

Conversely, different results were obtained when the TATA box was removed. Indeed, embryos microinjected with the +20bp(ΔTATA)GFP construct did not show any GFP expression (Fig 2B).

**Fig 3:**
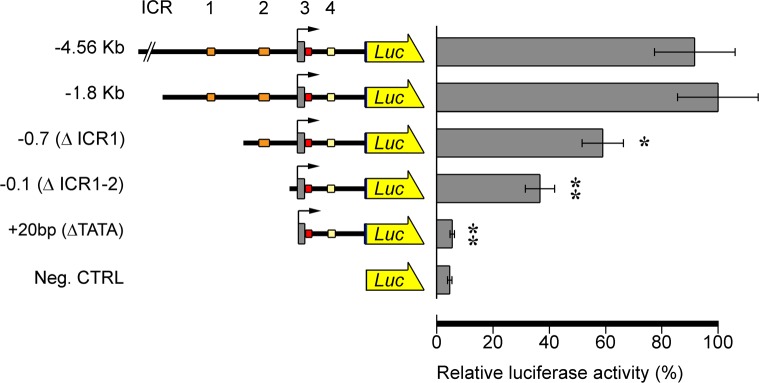
Enhancer functions of ICR1 and ICR2. Upstream deletions: luciferase activity progressively decreases after ICR1 and ICR2 sequential deletions. Left: schematic pictures of luciferase reporter constructs. Structure and conventions are the same as in Fig 1, except that the arrowed yellow box represents the Luc reporter gene. Right: luciferase activity measured at gastrula stage (24 hours post fertilization-hpf). The activity of the -1.8KbLuc construct is defined as 100%. Data are expressed as means ± standard deviation (SD) of triplet measurements of at least three independent experiments. Asterisks denote statistical significance: ** P value ≤ 0.0021, * P value = 0.0122.

To confirm all of these qualitative results, we performed quantitative targeted gene transfer assays using the luciferase reporter gene. Progressive 5’ deletions, from the recombinant clone Pl-Tuba1a-Luc to -1.8Kb (-1.8KbLuc construct) did not show any statistically significant difference, confirming that regulatory modules are not located in the region between -4.5 and -1.8 Kb (Fig 3). Differently, microinjections performed using the -0.7(ΔICR1)Luc construct showed a significant reduction of luciferase activity with respect to -1.8KbLuc (about 60% residual relative luciferase activity). A very large reduction was measured after microinjecting the -0.1(ΔICR1-2)Luc construct (about 40% residual relative luciferase activity, see Fig 3). Otherwise, gene transfer assays performed by microinjecting the +20bp(ΔTATA)Luc construct showed luciferase activity similar to negative controls (Fig 3).

All these results suggest that ICR1 and 2 may play a role as enhancers of *Pl-Tuba1a* gene expression, also confirming the functional role of the TATA box. Additionally, they provide evidence of the presence of regulatory modules in the first intron which may act activating the expression in the neurogenic territories.

### Functional analysis of interspecific conserved regions in the intron

While our results indicated that the first intron may contain regulatory modules activating and/or enhancing *Pl-Tuba1a* expression, it is also true that gene regulation is often controlled by quantitative, temporal and/or spatial regulatory modules that may be variously organized and potentially overlapping. Thus, we continued to use the double GFP/Luc approach.

**Fig 4:**
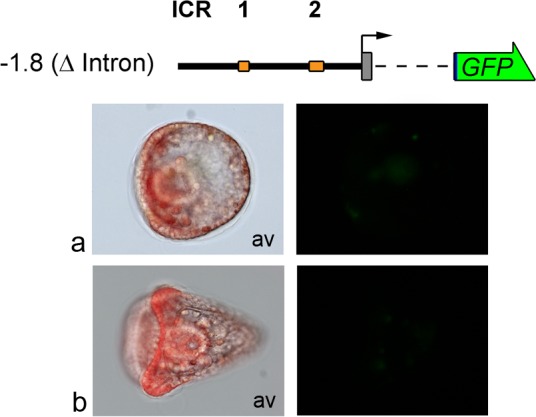
Transgene loss of expression by intron deletion. Embryos were microinjected with the -1.8(ΔIntron)GFP construct. Left: triple-merged images (bright-field, GFP fluorescence and Texas Red fluorescence) proving embryos are microinjected. Right: GFP fluorescence images. Structure and conventions are the same as in Fig 1. (a) Gastrula stage; (b) Pluteus stage. Av: animal view.

**Fig 5:**
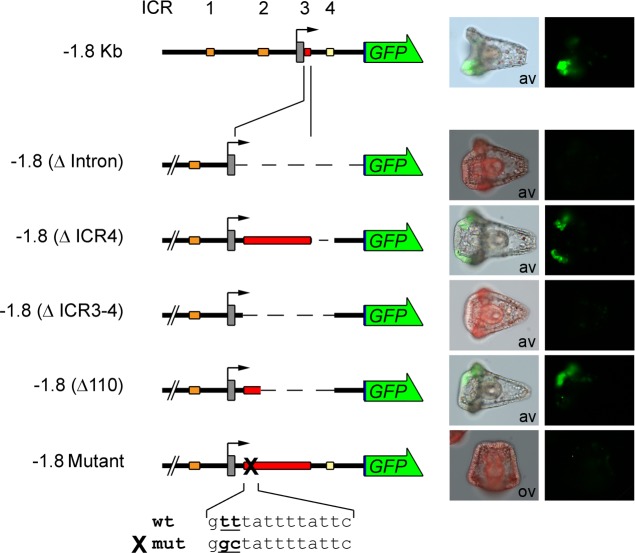
ICR3 and ICR4 function: spatial expression. In these construct depictions, ICR3 was enlarged to show deletion details (not to scale). Left: schematic pictures of GFP reporter constructs. Structure and conventions are the same as in Fig 1. Right: merged fluorescence and bright-field images or triple-merged images and fluorescence images from microinjected pluteus stage embryos are shown to observe GFP localization. × 20 magnification. Av: animal view; ov: oral view.

**Fig 6:**
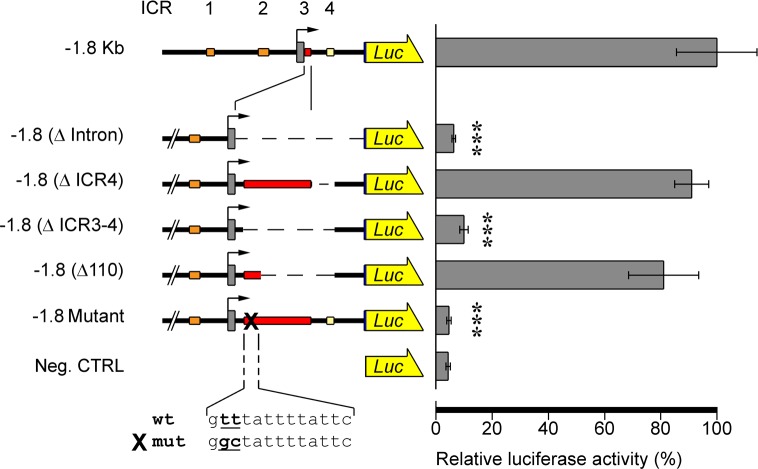
ICR3 and ICR4 function: quantitative analysis. In these construct depictions, ICR3 was enlarged to show deletion details (not to scale). Left: schematic pictures of luciferase reporter constructs. Structure and conventions are the same as in Fig 2. Right: luciferase activity measured at gastrula stage (24 hpf). The activity of the -1.8KbLuc construct is defined as 100%. Data are expressed as means ± SD of triplet measurements of at least three independent experiments. Asterisks denote statistical significance: *** P value < 0.0005.

Initially, in order to study the functional role of the intron, we performed gene transfer experiments using defective constructs in the whole first intron (from +96 to +898), which is localized after the tubulin start codon. Although this -1.8(ΔIntron)GFP construct encodes the tub-GFP fusion protein without splicing, microinjected embryos did not show any reporter expression during development, as shown in Figs 4 and 5. This result was confirmed by corresponding -1.8(ΔIntron)Luc construct microinjections (Fig 6). Indeed, intron removal dramatically affected luciferase activity, with results similar to negative controls.

Interestingly, the intron contains ICR3 and ICR4; therefore, to validate their involvement in *Pl-Tuba1a* regulation, we dissected intron sequences by microinjecting several GFP constructs lacking one or both ICRs. Obviously, because the intron contains important elements for transcript maturation whose mishandling could impair the proper functionality of the process, we took care to maintain all of the splice sites (5′ donor, 3′ acceptor and branch point) in the planning of the constructs.

Embryos microinjected with the -1.8(ΔICR4)GFP (deletion from +206 to +483) showed reporter expression similar to -1.8KbGFP, used as a positive control. Indeed, fluorescence appeared from the blastula stage (data not shown) to the pluteus stage, when it was localized in the ciliary band and the ganglia (Fig 5) as the endogenous transcript. Similarly, luciferase activity from embryos expressing -1.8(ΔICR4)Luc was comparable to the activity measured when the -1.8KbLuc construct was used (Fig 6). Thus, it is reasonable to suppose that the critical regulatory sequences are located in ICR3.

ICR3 contains the TATA box (from -29 bp to -22 bp), the first exon (from +1 bp to +95 bp) and 95 bp of the intron sequence. We have already demonstrated the pivotal role of the TATA box; additionally, an *in silico* analysis for transcription factor (TF) binding sites suggested the presence of putative consensus sequences located between +100 and +190 bp. Therefore, we extended intron deletion to +100 bp (deletion from +100 to +483), obtaining the -1.8(ΔICR3-4)GFP/Luc constructs.

The -1.8(ΔICR3-4)GFP injected embryos failed to express GFP in a manner that resembled whole intron deletion (Fig 5). The same results were obtained from the quantitative analysis of the corresponding Luc assays (Fig 6).

These results, then, suggest a transcriptional regulatory role for the region from +100 bp to +206 (ICR3 core).

### Detailed analysis of the ICR3 core and site-directed mutagenesis

**Fig 7:**
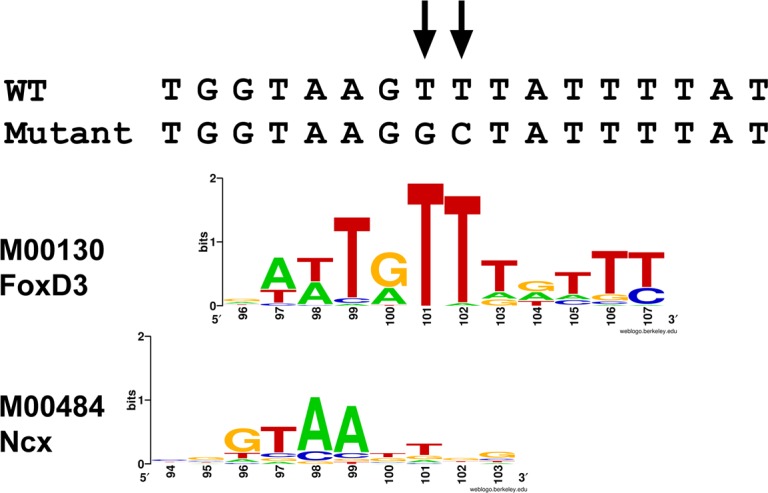
Transcription factor binding sites in the sequence necessary for proper *Pl-Tuba1a* expression. Top: wild-type and mutant construct sequences from +94 to +110 bp. Arrows indicate specific nucleotide changes generated by site-directed mutagenesis (T_101_ to G and T_102_ to C). Center and bottom: FoxD3 matrix logo (M00130) and Ncx (M00484) matrix logo aligned to sequences. Logos were generated using WebLogo [30].

In order to analyze the ICR3 core, new deletion constructs were built. In particular, microinjected constructs named -1.8(Δ110)GFP/Luc (deletion from +110 to +483), containing only 10 bp in addition to the non-functional -1.8(ΔICR3-4) construct, again showed the ability to drive the expression of the reporter genes in the apical organ and in the ciliary band (see Figs 5 and 6). Thus, we looked for a putative binding site of TFs located in the sequence near +100 bp. Both the Ncx and forkhead box (Fox) classes of TFs (best match of FoxD3 matrix is shown in Fig 7) could bind to this region. After a detailed match analysis (i.e. position, score), we decided to mutagenize two thymine residues in the Fox binding site. Therefore, by site-specific mutagenesis, we obtained a mutant clone (-1.8 Mutant) which differed from the wild-type by two base changes at positions 101–102 (T_101_ to G and T_102_ to C). Embryos microinjected with the mutant construct showed neither GFP expression nor luciferase activity (see Figs 5 and 6). Thus, these results suggest that this binding site is necessary to drive the proper expression of the *Pl-Tuba1a* gene.

## Conclusions

The sea urchin embryo is an excellent system for identifying the regulatory activities that coordinate neurogenesis events. It has also been shown that the gene products essential for neuroectoderm patterning in the deuterostome lineage, which includes the sea urchin, similarly act in other organisms and are also necessary for patterning the nervous system in chordates [31–33].

At the beginning of sea urchin embryogenesis, two neurogenic regions are specified by separate signals, including Wnt and TGFβ: the ANE and the more posterior CBE [12, 34]. ANE contains serotonergic and non-serotonergic neurons, as well as some cells that produce long, immotile cilia which might have a sensory function, whereas the CBE is predominantly composed of specialized ciliated cells and functions as a swimming and feeding organ. Interestingly, the apical organ was recently considered the central integrating component of the nervous system and the ciliary band a peripheral component that responds to stimuli and controls ciliary activity [9]. The *Pl-Tuba1a* gene, encoding an alpha tubulin isotype that might be used for primary cilia axoneme and for axon microtubules, is expressed in both the ANE and CBE regions [22].

In this study, we show that a genomic region of about 2.6 Kb of *Pl-Tuba1a* can recreate the spatio-temporal pattern of endogenous *Pl-Tuba1a* gene expression. It is well known that the analysis of ICRs is a useful tool for investigating *cis*-regulatory elements [35]. Thus, in order to look for *cis*-regulatory sequences in the *Pl-Tuba1a* gene, we have previously compared *P*. *lividus* and *Strongylocentrotus purpuratus tuba1a* orthologues. Using this strategy, we found six ICRs, and here we show that three of them have a regulatory role [26].

In particular, the data presented in this paper show that proper *Pl-Tuba1a* gene expression is enhanced by two upstream regions (ICR1 and 2) containing several TF binding sites, including a Fox consensus sequence.

The removal of ICR1 and 2 causes a decrease in *Pl-Tuba1a* expression level, suggesting that these modules may act by boosting *Pl-Tuba1a* gene expression in the ANE and CBE regions of the sea urchin embryo. Moreover, *Pl-Tuba1a* expression depends on a very short sequence spanning the transcription start site that contains a TATA box and a binding site for some members of the Fox transcriptional regulator family (FoxD, Q, J, etc.) whose consensus binding sequences are extremely similar [36]. Fox TFs bind DNA through a winged helix domain and play a pivotal role in differentiation and specification events, including the maturation process of neurons [37].

Fox TFs have also been extensively studied in other systems (mouse, frog, zebrafish, fruit fly). For instance, it has been demonstrated that FoxJ1 is required to regulate a cohort of ciliary genes to make motile cilia [38, 39], and that FoxD3 is required for neural crest specification [40, 41]. FoxA1 participates in stimulating the neuronal differentiation of pluripotent stem cells and activates the neural *betaIII tubulin* gene [42]; moreover, FoxA1 and FoxA2 are crucial for the specification, differentiation and maintenance of dopamine neurons during mouse embryonic development [43].

Interestingly, FoxD3 and FoxA1 are believed to scan chromatin for enhancers with forkhead motifs and to trigger their transcriptional competency through initial chromatin decompaction. According to this specific role, they have been designated “pioneer” TFs [36, 44]. Notably, FoxD3 pioneer factor occupancy can modulate the local epigenetic state of chromatin, serving as a placeholder until the later appearance of FoxA1 during mouse gastrulation [45].

It is noteworthy that a putative Fox binding site is located in ICR3 of *Pl-Tuba1a* and that its removal (by deletion or mutagenesis) impairs expression, suggesting a fundamental role for a Fox family member for *Pl-Tuba1a* activation during neural structure development.

In the purple sea urchin (*S*. *purpuratus*), 22 *fox* genes have been identified, and it has been shown that *foxD*, *foxJ1* and *foxQ2* are expressed in all or in a subset of cells derived from what is initially the animal pole of the embryo, and in the ciliary band by the end of gastrulation [43, 44]. In particular, FoxQ2, that at the gastrula stage is an animal pole marker, plays an essential role in the formation of the apical tuft cilia and is required for the development of serotonergic neurons [45–51]. Moreover, *foxG* (also known as Brain factor1) is expressed in the periphery of the ventral ectoderm at mesenchyme blastula, then strictly in the ciliary band [52].The finding that FoxD3 performs a priming activity is in good agreement with the previously demonstrated existence of developmentally dependent changes of the *Pl-Tuba1a* gene epitype. We have indeed shown that the *Pl-Tuba1a* gene begins to be expressed only when specific histone modifications (H3K9Ac, H3K4me3) induce an accessible chromatin conformation of the core promoter [27].

Finally, it is remarkable that the expression profile of *Pl-Tuba1a* seems to overlap with that of *foxJ1/Q2* and *foxG*, suggesting a possible sequential enhancement/priming role of these factors in the sequential *Pl-tuba1a* expression during the differentiation of the animal plate and ciliary band.

**Fig 8:**
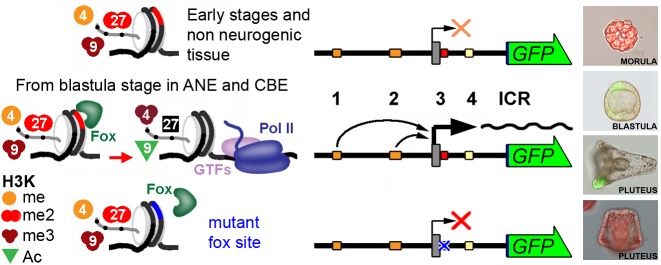
A summary of the transcription regulation of the *Pl-Tuba1a* gene. Chromatin modifications occurring on the *Pl-Tuba1a* promoter, according to [27] are shown. Transgenic wild type and mutagenized constructs and their expression during embryo development are also shown. See text for details.

Therefore, it could be hypothesized that the binding site in ICR3 likely represents a holder platform for a Fox pioneer factor inducing both early specific neurogenic activation and later expression maintenance. Accordingly, a model in which FoxQ2 initiates an anterior patterning centre that implements correct size and positions of ANE structures was recently proposed [53]. Additionally, after chromatin remodeling and increased accessibility, Fox member(s) bound to ICR2 might enhance *Pl-Tuba1a* gene expression (Fig 8).

## Supporting Information

S1 Table Oligonucleotides used as PCR primers for construct preparation. Underlined sequences indicates restriction enzyme recognition sites.
(DOCX)

S1 Fig Expression of the GFP transgene constructs in *P*. *lividus* sea urchin embryo at the pluteus stage. At left is a schematic structure (drawn to scale) of the Pl-Tuba1a-GFP reporter constructs. The bent arrow indicates the transcription start site. A grey box represents the first exon (5’UTR and ATG start codon). Downstream of the first exon there are the first intron and two codons of the second exon. Coloured boxes indicate the four ICRs. For sake of simplicity, only the section/segment of ICR3 inside the intron is shown. The arrowed green box represents the GFP reporter gene cloned in frame with the alpha tubulin codons. At right: (left column) merged fluorescence and bright-field images or triple-merged images (bright-field, GFP fluorescence and Texas Red fluorescence, last construct); (right column) GFP fluorescence images from microinjected embryos (animal views). × 20 magnification.
(TIF)
