# Supplementary material for: An Intronic cis-Regulatory Element Is Crucial for the Alpha Tubulin Pl-Tuba1a Gene Activation in the Ciliary Band and Animal Pole Neurogenic Domains during Sea Urchin Development
Source: PLoS One. 2017 Jan 31;12(1):e0170969. doi: 10.1371/journal.pone.0170969 (PMC5283682; doi:10.1371/journal.pone.0170969)
Supplement: S1 Table — Underlined sequences indicates restriction enzyme recognition sites. (DOCX) [file pone.0170969.s001.docx]

| 5’ deletion constructs | | 5’-3’ Sequences |
| --- | --- | --- |
| -4.2 Kb | Forward | GCACAAGCTTGTCTATGGCAGCGTT |
| -3.9 Kb | Forward | GCACAAGCTTTCCAGTACATTCCCG |
| -3.5 Kb | Forward | GCACAAGCTTGCGGTGACAAACACATTC |
| -2.6 Kb | Forward | GCACAAGCTTGTCGCAACATATTCG |
| -1.8 Kb | Forward | GCACAAGCTTGCGGACTTTTTGGTATTAGG |
| -1.1 (ΔICR1) | Forward | GCACAAGCTTACCACTAGCAGTGTC |
| -0.9 (ΔICR1) | Forward | ACGCAAGCTTGATTATGGTGGGGTGTTGCC |
| -0.7 (ΔICR1) | Forward | GCGTAAGCTTGTCGACAGATTTTCTAACC |
| -0.3 (ΔICR1-2) | Forward | ACGTAAGCTTGGGACTGCAGGACATGATAGTAG |
| -0.2 (ΔICR1-2) | Forward | ACGCAAGCTTCAAATCGTACATAACACGCC |
| -0.1 (ΔICR1-2) | Forward | ACGCAAGCTTCGGTTGACAACCGCTGACGT |
| +20 bp(ΔTATA) | Forward | ACGCAAGCTTCACATCGCACGTCGAACGGCA |
| Shared | Reverse | ACGCAAGCTTGTTGGCCGATTCATTAATGC |
| Internal deletion constructs | | **5’-3’ Sequences** |
| Shared 1 (-1.8 kb) | Forward 1 | ACGCAAGCTTGCGGACTTTTTGGTATTAGG |
| -1.8 (Δ Intron) | Reverse 1 | ACCGGGTACCCTTCACGCATGATGATACATTATTCGAATTCG |
|  | Forward 2 | CTCGGTGGAGGGAGAATAG |
| -1.8 (ΔICR4) | Reverse 1 | GCTCTAGACGTTTGTAGATTCGTCATCTC |
|  | Forward 2 | GCTCTAGAGGAATTGAGTTGTGACTGAC |
| -1.8 (ΔICR3-4) | Reverse 1 | GCTCTAGATTACCATGATGATACATTATTCG |
|  | Forward 2 | GCTCTAGAGGAATTGAGTTGTGACTGAC |
| -1.8 (Δ110) | Reverse 1 | GCTCTAGATAAAATAAACTTACCATGATG |
|  | Forward 2 | GCTCTAGAGGAATTGAGTTGTGACTGAC |
| Shared | Reverse 2 | ACGCAAGCTTGTTGGCCGATTCATTAATGC |
| -1.8 Mutant | Forward | GAATTCGAATAATGTATCATCATGGTAAGGCTATTTTATTCACATCTGCCTCATTCTTTT |
|  | Reverse | GAATGAGGCAGATGAAAATGAATAAAAATAGCCTTACCATGATGATACATTATTCGAATTC |
| GFP-Luc cassette exchange | Forward | GGGGTACCCTGTTGGTAAAATGGAAGACGC |
|  | Reverse | GGGGTACCAAGCTTGGACAAACCAGAACTAGAATGC |
