# Supplementary figures and images for: An Intronic cis-Regulatory Element Is Crucial for the Alpha Tubulin Pl-Tuba1a Gene Activation in the Ciliary Band and Animal Pole Neurogenic Domains during Sea Urchin Development
Source: PLoS One. 2017 Jan 31;12(1):e0170969. doi: 10.1371/journal.pone.0170969 (PMC5283682; doi:10.1371/journal.pone.0170969)

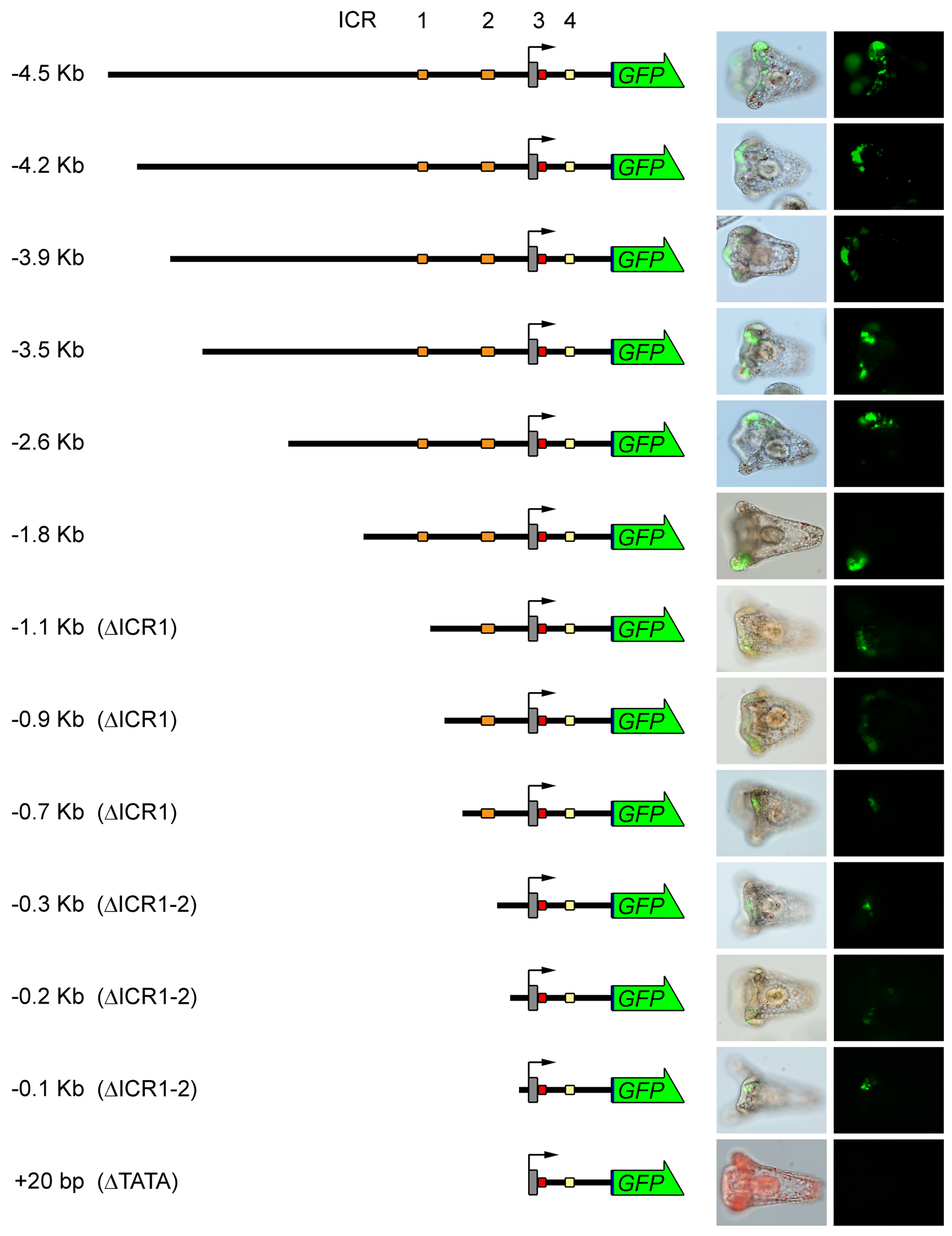

Supplement: S1 Fig — At left is a schematic structure (drawn to scale) of the Pl-Tuba1a-GFP reporter constructs. The bent arrow indicates the transcription start site. A grey box represents the first exon (5’UTR and ATG start codon). Downstream of the first exon there are the first intron and two codons of the second exon. Coloured boxes indicate the four ICRs. For sake of simplicity, only the section/segment of ICR3 inside the intron is shown. The arrowed green box represents the GFP reporter gene cloned in frame with the alpha tubulin codons. At right: (left column) merged fluorescence and bright-field images or triple-merged images (bright-field, GFP fluorescence and Texas Red fluorescence, last construct); (right column) GFP fluorescence images from microinjected embryos (animal views). × 20 magnification. (TIF) [file pone.0170969.s002.tif]
